# Ultrasonic-Assisted Extraction, Structural Characterization, Chain Conformation, and Biological Activities of a Pectic-Polysaccharide from Okra (*Abelmoschus esculentus*)

**DOI:** 10.3390/molecules25051155

**Published:** 2020-03-05

**Authors:** Xi-Rui Nie, Yuan Fu, Ding-Tao Wu, Ting-Ting Huang, Qin Jiang, Li Zhao, Qing Zhang, De-Rong Lin, Hong Chen, Wen Qin

**Affiliations:** College of Food Science, Sichuan Agricultural University, Ya’an 625014, China; niexrachel@163.com (X.-R.N.); yuanffuy@163.com (Y.F.); HTT84640527@163.com (T.-T.H.); xinchenggui@163.com (Q.J.); zhaoli0608@126.com (L.Z.); zhangqing@sicau.edu.cn (Q.Z.); lindr2018@sicau.edu.cn (D.-R.L.); chenhong945@sicau.edu.cn (H.C.)

**Keywords:** okra polysaccharides, structural characteristics, chain conformation, biological activities

## Abstract

The purpose of this study was to better understand the chemical characteristics and chain conformation of okra polysaccharides extracted by ultrasonic-assisted extraction. A pectic-polysaccharide, named OPP-D, was obtained, which was mainly composed of rhamnose, galacturonic acid, and galactose with a molar ratio of 1.01:1.00:2.31. Combined with NMR analysis, -4)-α-d-GalA*p*-(1,2,4)-α-l-Rha*p*-(1- were identified as the backbone with galactan side chains substituted partly at O-4 of Rha*p*. Molecular weight and radius of gyration of OPP-D were determined as 2.19 × 10^5^ Da and 27.0 nm, respectively. OPP-D was determined as an air-core sphere with branching chains in 0.9% NaCl solution by high-performance size-exclusion chromatography coupled with multi-angle laser light scattering and dynamic light scattering for the first time. Moreover, OPP-D exhibited typical shear-thinning behavior. In addition, OPP-D exhibited remarkable in vitro antioxidant activities and prebiotic activities, while the relatively high molecular weight, high degree of esterification, high content of uronic acids, and highly branched globular conformation of OPP-D might contribute to its in vitro anti-diabetic activities and binding capacities. Results can contribute to a better understanding of the structure–bioactivity relationship of OPPs, and OPP-D has great potential applications in the functional food and pharmaceutical industries.

## 1. Introduction

*Abelmoschus esculentus* (L.) Moench, a delicious vegetable known as okra, has been used worldwide as a functional food and folk medicine for many years [1]. It is native to Africa and has been cultivated in China [2,3]. Previous studies have reported that okra has abundant bioactivities, including anti-fatigue [2], antioxidant [4], anti-hyperglycemic, and anti-diabetic effects [5]. Generally, one of the major health-promoting properties in okra is referred to as polysaccharides [6]. Okra polysaccharides (OPPs) extracted by hot water extraction have been investigated. Generally, OPPs have been reported to possess relatively high molecular weights ranging from 2.99 × 10^6^ to 5.94 × 10^6^ Da [7,8]. The relatively high content of uronic acids in OPPs confirms the presence of pectic-polysaccharides [9]. Moreover, the major constituent monosaccharides of OPPs are determined as rhamnose, galacturonic acid, galactose, and arabinose [10]. Nevertheless, the chain conformation of OPPs and their structure–bioactivity relationships are still insufficient.

Generally, extraction methods have noticeable influences on the physicochemical characteristics and bioactivities of OPPs [11]. Ultrasonic-assisted extraction (UAE), as a green extraction method, has already been widely exploited for accelerating the extraction processes of polysaccharides [12]. UAE is utilized due to the acoustic cavitation to destroy cell walls, reduce particle sizes, and enhance the contact between solvents and targeted compounds [13,14]. UAE has several advantages, such as short extraction time, moderate solvent requirements, and minimal impacts on bioactivities [4]. Previous studies have investigated the extraction optimization and antioxidant activities of OPPs extracted by UAE, which confirm that the UAE method can be an efficient method to extract OPPs with high antioxidant activities [4]. However, the detail chemical structures and chain conformation of OPPs extracted by UAE, as well as their structure–bioactivity relationships are still unknown.

Therefore, in order to well understand the detail chemical structures and chain conformation of OPPs and improve the applications of OPPs in the functional food and pharmaceutical industries, the purification, structural characteristics, chain conformation, and bioactivities of OPPs extracted by UAE were investigated.

## 2. Results and Discussion

### 2.1. Physicochemical Characteristics

#### 2.1.1. Chemical Compositions of OPP-D

The extraction yield of OPPs extracted by UAE was 13.99% ± 0.52%, which was comparable to the extraction yields of OPPs extracted by microwave-assisted extraction (MAE), hot water extraction (HWE), and pressurized water extraction (PWE) previously reported [11]. The extraction time of UAE was much shorter than those of HWE and PWE, and the extraction temperature of UAE was also lower than those of HWE, PWE, and MAE, which suggested that the UAE method was a more efficient and green method.

Moreover, the chemical compositions of the purified OPP (OPP-D) were determined. The content of total polysaccharides in OPP-D was determined to be 92.85% ± 2.27%, and the content of proteins was 0.33% ± 0.07%, which suggested that polysaccharides were the main biological components in OPP-D. Moreover, the content of uronic acids in OPP-D was determined as 33.35% ± 2.38%. In general, such high content of uronic acids in natural polysaccharides might lead to strong antioxidant activities, digestive enzyme inhibitions, and binding properties [9,15,16]. However, the content of total uronic acids in OPP-D extracted by UAE was significantly lower than that of OPPs extracted by MAE, HWE, and PWE [11], which might be attributed to the degradation of homogalacturonan (HG) in OPP under the ultrasonic condition [14,17].

#### 2.1.2. Constituent Monosaccharides of OPP-D

**Figure 1:**
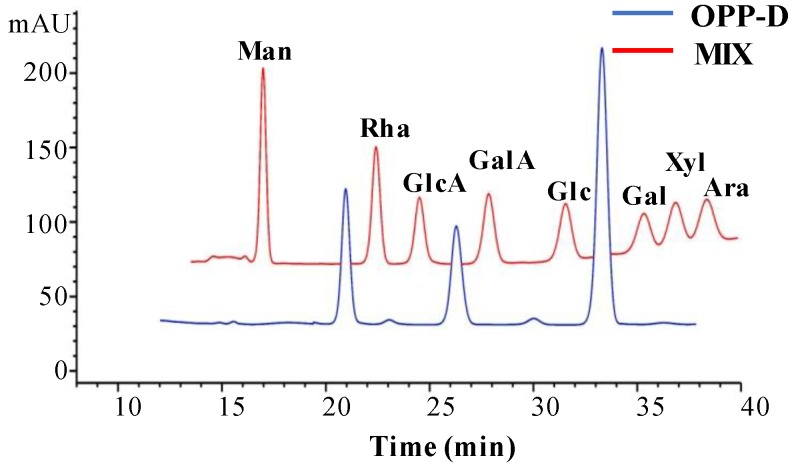
High-performance liquid chromatography profile of purified okra polysaccharides (OPP-D). Man, mannose; Rha, rhamnose; GlcA, glucuronic acid; GalA, galacturonic acid; Glc, glucose; Gal, galactose; Xyl, xylose; Ara, arabinose.

Monosaccharides are the natural basic units that determine the unique structures and properties of polysaccharides [11]. The monosaccharide composition of OPP-D was investigated on the basis of PMP pre-column derivation by high-performance liquid chromatography (HPLC). As shown in Figure 1, the major constituent monosaccharides of OPP-D were measured as rhamnose (Rha), galacturonic acid (GalA), and galactose (Gal). The molar ratio of Rha, GalA, and Gal in OPP-D was determined to be about 1.01:1.00:2.31, respectively. This demonstrated that OPP-D was a pectic-polysaccharide. In addition, the close molar ratio of Rha and GalA indicated that they might be the residues building the backbone, while galactose might be the residue constructing the side chains as it had the highest molar ratio [10,18].

#### 2.1.3. FT–IR Spectra and Esterification Degree of OPP-D

**Figure 2:**
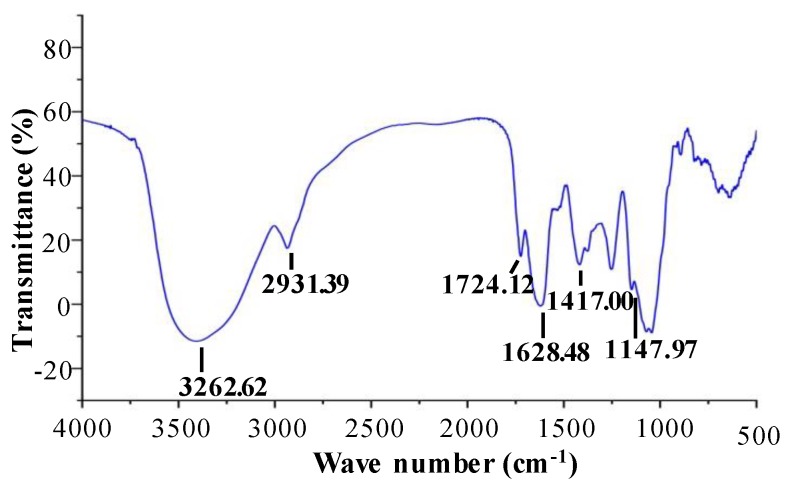
FT–IR spectra of OPP-D.

In order to determine the structural features of OPP-D, the FT–IR spectra were measured. As shown in Figure 2, the broad band at 3262.62 cm^−1^ corresponds to the hydroxyl group stretching vibration [19]. The absorption peak at 2931.39 cm^−1^ is attributed to the stretching vibration of C-H asymmetric [19]. Then, the absorption peaks at 1724.12 cm^−1^ and 1628.48 cm^−1^ are owing to esterified groups of the C=O and COO- stretching vibration, which indicates the presence of uronic acids [11]. Results were consistent with the constituent monosaccharides of OPP-D. The absorption peaks at 1417.00 cm^−1^ and 1147.97 cm^−1^ are owing to the C-H or O-H and C-O-C bending vibration, which indicates the existence of -OCH3 [9]. Furthermore, the degree of esterification (DE) of OPP-D was calculated as 22.81% ± 0.85%. Previous studies have reported that the high DE in natural polysaccharides may lead to high inhibition of digestive enzymes [20], while the low DE may lead to high antioxidant activities [19,21]. Furthermore, results suggested that the chemical structure of OPP-D was similar to the chemical structures of OPPs extracted by MAE, HWE, and PWE, but the DE in OPP-D was lower than the DE in OPP extracted by HWE and PWE [11]. This might be related to the degradation of OPP-D under the ultrasonic condition [17].

#### 2.1.4. Structure Prediction of OPP-D by NMR Analysis

**Figure 3:**
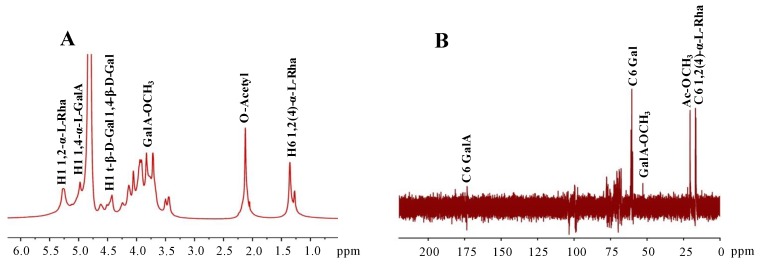
^1^H (**A**) and ^13^C (**B**) NMR spectra of OPP-D.

NMR spectra were recorded in order to reveal the precise structural information of OPP-D. 1D NMR spectra, including ^1^H and ^13^C analyses, were shown in Figure 3A,B. The ^1^H signal at 4.80 ppm belongs to D_2_O. In combination with the constituent monosaccharides of OPP-D, the abroad peaks at 1.26 and 1.35 ppm are attributed to the H-6 of 1,2-α-l-Rha and 1,2,4-α-l-Rha, respectively [18]. The signal at 5.29 ppm is also attributed to H-1 of 1,2-α-l-Rha [18]. The signal at 2.10 ppm corresponds to the existence of acetyl groups [22]. The existence of 3.82 ppm is the signal of methyl esters connecting to carboxyl groups of D-GalA [8]. The abroad peaks between 4.45 and 4.65 ppm indicate the existence of t-*β*-d-Gal and 1,4-*β*-d-Gal [23]. The abroad peak at 4.96 ppm is attributed to the H-1 of 1,4-α-l-GalA [23].

The ^13^C NMR spectra (Figure 3B) of OPP-D showed the C-6 signals for 1,2-α-l-Rha and 1,2,4-α-l-Rha at 16.61 and 16.77 ppm [5]. The presence of 20.63 ppm can be readily attributed to the methyl of acetyl groups [22]. The signals at around 52.48 ppm correspond to the existence of a methyl group esterified carboxyl group of GalA [8,22]. The abroad peaks at around 60.46 ppm are attributed to the C-6 of Gal [10]. The peaks at around 174.1 ppm correspond to the C-6 of un-esterified carbonyl groups of D-GalA [22].

Finally, combined with the constituent monosaccharides and FT–IR spectra of OPP-D as well as the published studies [10,18], these results suggested that the probable structure of OPP-D was -4)-α-d-GalA*p*-(1,2,4)-α-l-Rha*p*-(1- as backbone and 1,4-*β*-d-galactan as side chains.

#### 2.1.5. Molecular Weight and Chain Conformation of OPP-D

**Figure 4:**
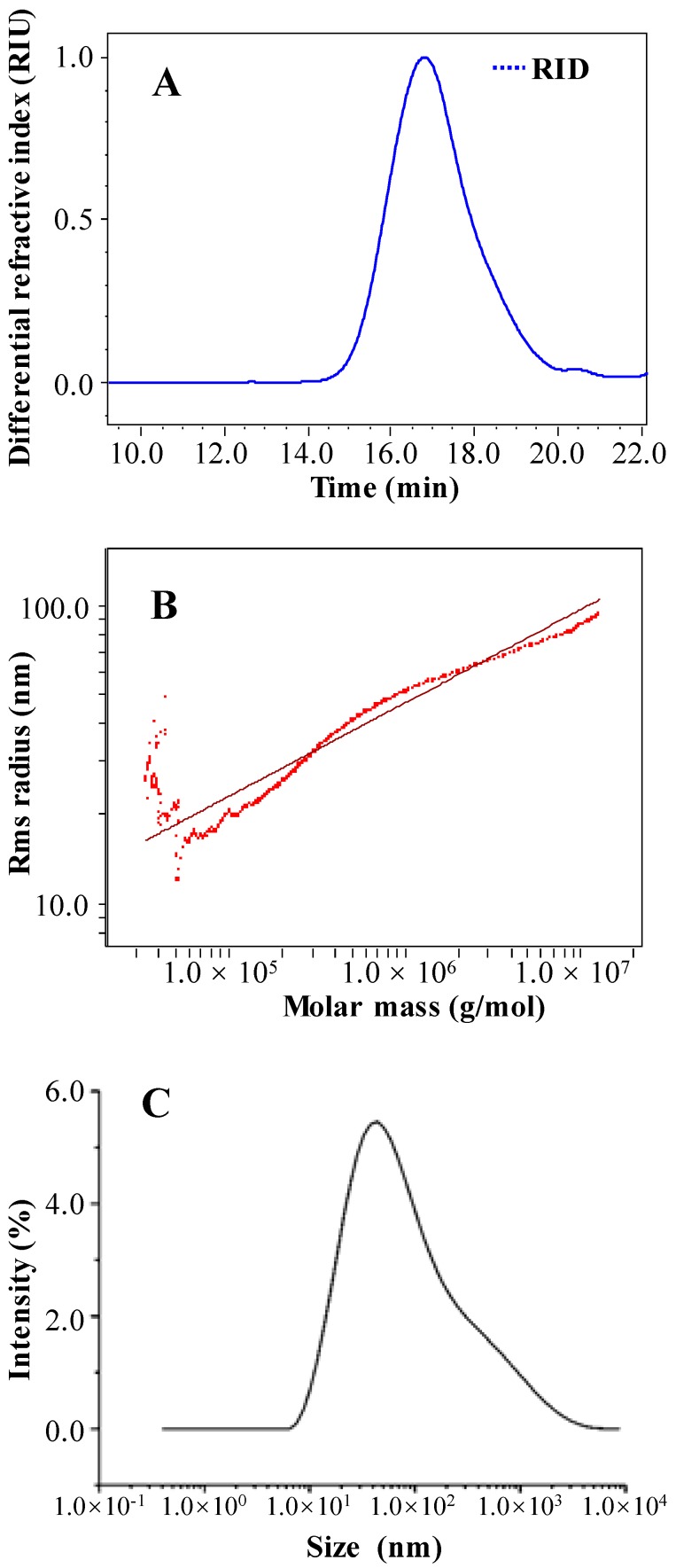
High-performance size-exclusion chromatogram (**A**), dependence of radius of gyration on molar mass (**B**), and hydrodynamic radius (**C**) of OPP-D.

Usually, the bioactivities of polysaccharides are connected with their molecular weight and chain conformation [24]. Thus, the molecular weight, polydispersity (*M_w_*/*M_n_*), the radius of gyration (*R_g_*), and chain conformation of OPP-D were investigated. Figure 4A showed that OPP-D was a symmetrical polysaccharide fraction, which suggested that OPP-D was a homogeneous pectic-polysaccharide. Combined with the high content of total polysaccharides and low content of protein, results confirmed that OPP-D was a highly purified polysaccharide. The molecular weight of OPP-D was determined as 2.19 × 10^5^ (±0.40%) Da, and the *R_g_* of OPP-D was measured at 27.0% ± 3.9% nm. The molecular weight of OPP-D was significantly lower than those of polysaccharides extracted by HWE, PWE, and MAE [11], which might be associated with the degradation of polysaccharide by UAE [14,17]. In addition, the polydispersity of OPP-D was 1.76% ± 2.11%, which demonstrated that OPP-D had a relatively broad molecular weight distribution.

The radius of gyration (*R_g_*) is a constant which can characterize the molecular size of the polymer, and *R_g_* can be calculated by the equation *R_g_* = k*M_w_*^v^ [25,26]. The index v can be calculated by the linear slope from the configuration diagram constructed by log (*R_g_*) to log (*M_w_*), and v = 0.33 means spherical molecules, ν = 0.5–0.6 means a random string, while ν = 1 means a rigid rod [24]. Figure 4B showed the relationship between molecular weights and radius of gyration. As calculated from Figure 4B, the index v was 0.32, which indicated that OPP-D existed as a spherical molecule in aqueous solution.

Moreover, the size of polysaccharides in solution can also be determined by dynamic light scattering (DLS), and *R_h_* can be calculated by the equation D = KT/6πηr [25]. As shown in Figure 4C, the *R_h_* of OPP-D in 0.9% NaCl solution was determined as 25.3 ± 0.1 nm. The structure sensitive parameter ρ can reflect the molecular conformation in solution, and ρ can be calculated by the equation ρ = *R_g_*/*R_h_* [26]. For a specific polymer solution, the value of ρ represents information of the chain conformation, structure, and polydispersity index of the polymer, and ρ = 0.7–0.8 means compact sphere, ρ = 1.0–1.1 means air-core sphere with branching chains, while ρ = 1.5–1.8 means curly chain without rules. The ρ of OPP-D was calculated to be 1.07, which meant that OPP-D existed an air-core sphere with branching chains in aqueous solution [24].

#### 2.1.6. Apparent Viscosity of OPP-D

**Figure 5:**
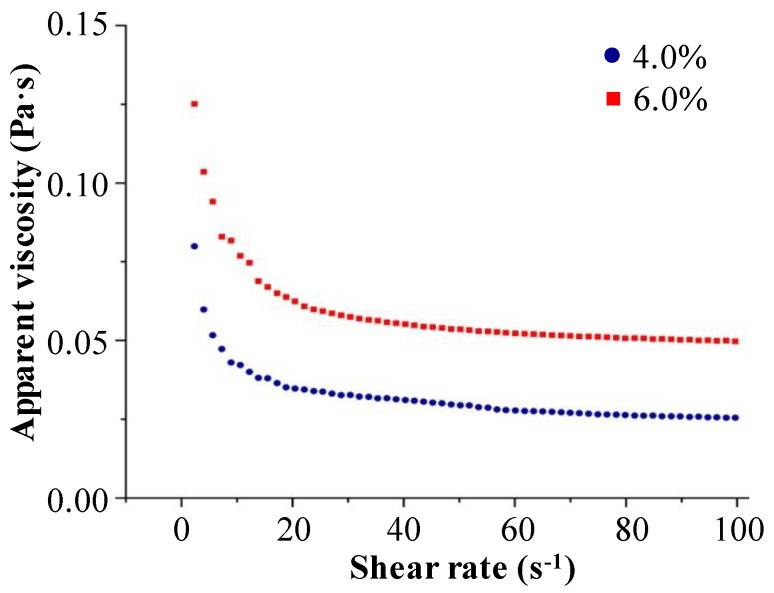
Dependence of apparent viscosity on the shear rate of OPP-D.

It is well believed that the apparent viscosity of polysaccharide is closely related to molecular characteristics [27]. The apparent viscosities of OPP-D solutions are shown in Figure 5. As shown in Figure 5, the apparent viscosities of OPP-D increased with the increase of concentration, which is similar to the previous study [9]. This property might be associated with the initiation of individual molecular overlapping and increasing the formation of intermolecular connections, resulting in limited alignment and stretching of the polymer chains, and increased the apparent viscosity [28]. In addition, the apparent viscosity of OPP-D decreased with increasing shear rate. The OPP-D solutions exhibited non-Newtonian shear-thinning behaviors at 0.01–50 s^−1^, while nearly Newtonian flow behavior at 50–100 s^−1^. For such shear-thinning behavior may mainly be associated with the untangling of the molecular chains in solution [28,29]. Moreover, compared to our previous study, OPP-D exhibited a significantly lower apparent viscosity than OPPs extracted by HWE [9], which mainly due to relatively lower molecular weight and wider polydispersity of OPP-D [30]. All these results suggested that OPP-D possessed typical shear-thinning behavior.

### 2.2. Antioxidant Activities of OPP-D

**Figure 6:**
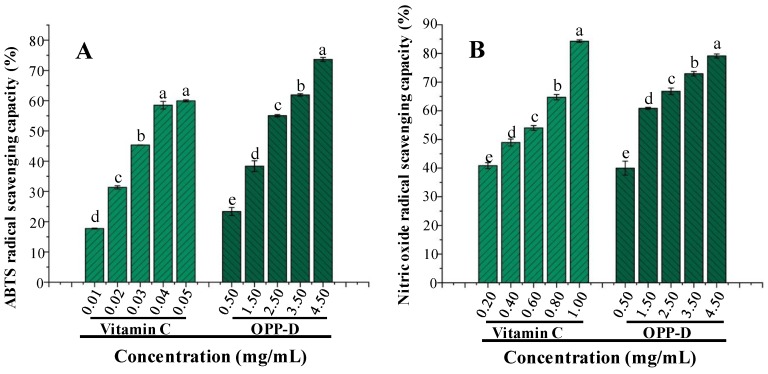
2,2′-Azino-bis (3-ethylbenzthiazoline-6-sulphonic acid) ABTS radical cation scavenging activity (**A**) and nitric oxide radical scavenging activity (**B**) of OPP-D. The error bars are standard deviations. Significant (*p* < 0.05) differences are shown by data bearing different letters (a–e). Statistical significances were carried out by ANOVA and Duncan’s test.

Previously reported studies have already reported that polysaccharides from okra possess remarkable antioxidant activities [9,11]. Therefore, the antioxidant activities of OPP-D were measured. The 2,2′-azino-bis (3-ethylbenzthiazoline-6-sulphonic acid) (ABTS) and NO radical scavenging activities of OPP-D are shown in Figure 6. As shown in Figure 6A,B, the ABTS and NO radical scavenging activities of OPP-D demonstrated a dose-dependent manner, respectively, and both reached the highest scavenging activities at 4.50 mg/mL, which indicated that OPP-D exhibited remarkable antioxidant activities. In brief, the IC_50_ values of ABTS and NO radical scavenging activities of OPP-D were evaluated as 2.47 mg/mL and 0.98 mg/mL, respectively. Additionally, compared to the positive control (vitamin C), OPP-D demonstrated good ABTS and NO radical scavenging activities. Moreover, the antioxidant activities of OPP-D were similar to OPPs extracted by MAE, but relatively higher than those of polysaccharides extracted by HWE and PWE from our previous study [11]. The longer ultrasonic exposure time tends to lower molecular weight and viscosity, shorter chain length, and more hydroxyl groups [31]. The antioxidant activities of natural polysaccharides are associated with their *M_w_*, uronic acids, and constituent monosaccharides [15,32]. Thus, the relatively high antioxidant activities of OPP-D may be owing to its high content of unmethylated galacturonic acid [9,11]. Results also suggested that OPP-D as a natural antioxidant had great potential applications in the food and medical industry.

### 2.3. In Vitro α-amylase and α-glucosidase Inhibitory Activities of OPP-D

**Figure 7:**
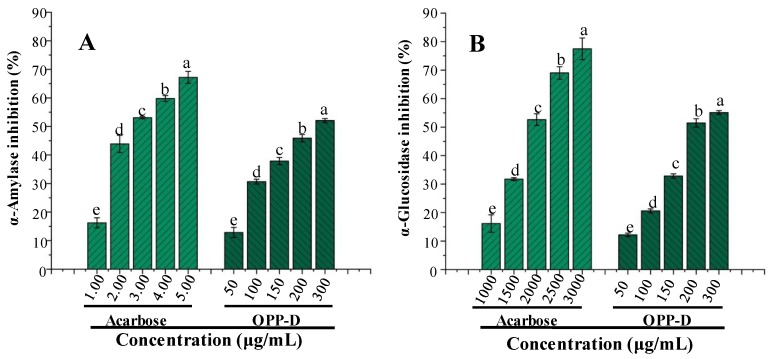
In vitro inhibitory activities on α-amylase (**A**) and α-glucosidase (**B**) of OPP-D. The error bars are standard deviations. Significant (*p* < 0.05) differences are shown by data bearing different letters (a–e). Statistical significances were carried out by ANOVA and Duncan’s test.

The major strategies for counteracting metabolic changes associated with hyperglycemia and type 2 diabetes are the inhibitions of α-amylase and α-glucosidase [33], and our previous studies have reported that OPPs possessed significant in vitro anti-hyperglycemic activity [9,11]. Therefore, in vitro α-amylase and α-glucosidase inhibitions of OPP-D were evaluated (Figure 7A,B). Results demonstrated that in vitro α-amylase and α-glucosidase inhibitions of OPP-D exhibited a dose-dependent manner, and OPP-D showed significant in vitro α-amylase and α-glucosidase inhibitions. The IC_50_ values of in vitro α-amylase and α-glucosidase inhibitions of OPP-D were determined as 223.77 μg/mL and 215.70 μg/mL, respectively. Compared with acarbose (IC_50_ = 2020.41 μg/mL), the in vitro α-glucosidase inhibition of OPP-D was significantly stronger. Furthermore, the in vitro digestive enzyme inhibitions of OPP-D were slightly lower than OPPs extracted by HWE, PWE, and MAE [9,11]. The inhibitions on the digestive enzyme of OPP-D were significantly higher than that of pectic-polysaccharides from *Annona squamosa* [34] and bitter gourd [32], which might be related to its relatively high *M_w_*, high DE, and high content of uronic acids [9,20]. Moreover, the highly branched globular conformation of OPP-D in aqueous solution may contribute to available active hydroxyl groups to exert the anti-diabetic activity [35]. Results also suggested that OPP-D as the treatment of type 2 diabetes had great potential applications in the food and medical industry.

### 2.4. In Vitro Binding Properties of OPP-D

Excessive absorption of bile acids, cholesterol, and fat may lead to obesity problems, which are related to diabetes cancer and cardiovascular disease [16], and our previous studies have reported that OPPs possess significant in vitro binding properties [9,11]. The fat, cholesterol, and bile acid-binding capacities of OPP-D were determined to be 1.47 ± 0.23 mg/mg, 19.34 ± 1.38 mg/g, and 39.61% ± 0.37%, respectively. Compared with the positive controls, OPP-D showed slightly stronger in vitro binding properties. Furthermore, OPP-D showed lower in vitro binding properties than OPPs extracted by HWE, PWE, and MAE. OPP-D showed similar in vitro binding properties to the *β*-glucans from Qingke [16], and higher than that of pectic-polysaccharides bitter gourd [32]. The in vitro binding properties of OPP-D might be related to its DE, *M_w_*, and molecular weight distributions [9,20]. Moreover, such high in vitro binding properties may also be attributed to the highly branched globular conformation of OPP-D and strong hydrogen bonds of carboxylic and hydroxyl groups [36]. Results suggested that OPP-D as functional food ingredients had good potential applications to prevent hypercholesterolemia and hyperlipidemia.

### 2.5. In Vitro Prebiotic Activities of OPP-D

Previous studies have reported that polysaccharides exert their health benefits called prebiotic activities in the human intestine, regulating the production of short-chain fatty acids (SCFAs) [37]. Short-chain fatty acids (SCFAs) are the main end products generated of undigestible carbohydrate fibers by bacterial fermentation, and may contribute to the acidic environment that can inhibit the growth of pathogens in the human gut, thereby altering the intestinal bacterial composition and improving the health of the host [38]. The effects of OPP-D on the growth of three *Lactobacilli* strains, including *L. acidophilus* CICC 6089, *L. rhamnosus* CICC 6133, and *L. rhamnosus* CICC 6151 were investigated to determine whether OPP-D was a potential substrate to be metabolized by colon microbiota. As shown in Table 1, the number of all tested *Lactobacilli* strains treated with OPP-D and the total SCFAs were significantly increased, respectively. These meant that OPP-D was not toxic to the assayed probiotics, whereas it was a good substrate for facilitating probiotic growth, and OPP-D could be utilized by probiotic bacteria to sustain survival and metabolic activities. Polysaccharides comprised of galactose may have typically displayed greater prebiotic activity [38,39], and the linkages of polysaccharides are also essential for prebiotic activities, which RG-I may have significant proliferative effects [37,40]. Additionally, notable increases in the OD_600_ were observed which ranged from 0.28 to 0.35, from 0.33 to 0.40, and from 0.32 to 0.40, respectively. Furthermore, similar results could be found in the production of SCFAs, and the total SCFAs ranged from 16.17 to 31.32 mM, from 10.28 to 20.81 mM, and from 54.38 to 90.98 mM, respectively. Results showed that all tested Lactobacilli strains exhibited a significantly dose-dependent manner, where a higher content of polysaccharides could have a better proliferative effect [41]. Moreover, the inulin, as for positive control, possessed greater prebiotic activities than OPP-D at the same concentration. Polysaccharides with lower *M_w_*, better water solubility, and lower viscosity may exert better prebiotic effect [37]. All these results indicated that OPP-D had good potential applications as functional food ingredients for prebiotic activity.

## 3. Materials and Methods

### 3.1. Material, Chemicals, and Lactobacillus Strains

Okra fruits were harvested at a commercial orchard in Chengdu, Sichuan, China. The samples were washed, hot-air-dried (75 °C and 12 h), smashed, screened, and stored at −20 °C. Acarbose, α-amylase (1000 U/mg), α-glucosidase (10 U/mg), arabinose (Ara), galactose (Gal), galacturonic acid (GalA), glucose (Glc), glucuronic acid (GlcA), mannose (Man), rhamnose (Rha), xylose (Xyl), 2,2′-azino-bis (3-ethylbenzthiazoline-6-sulphonic acid) (ABTS), 1-phenyl-3-methyl-5-pyrazolone (PMP), and 4-nitrophenyl *β*-d-glucopyranoside (pNPG) were all purchased from Sigma-Aldrich (St. Louis, MO, USA). A free cholesterol assay kit and DEAE Cellulose-52 were purchased from Solarbio (Beijing, China). *Lactobacillus acidophilus* CICC 6089, *Lactobacillus rhamnosus* CICC 6133, and *Lactobacillus rhamnosus* CICC 6151 were purchased from China Center of Industrial Culture Collection. All other chemicals and reagents used were of analytical grade.

### 3.2. Extraction and Preparation of OPPs

Extraction and preparation of OPPs were performed by the formerly reported method with minor modifications [11,19]. In brief, 40.0 mL of phosphate buffer solutions (50 mM, pH 6.0) were used to extract OPPs twice by ultrasonic-assisted extraction with an Ultrasonic Processor (650 W, 24 kHz, Scientz, Ningbo, China) at room temperature. The ultrasound extraction amplitude and time were set as 75% and 20 min, respectively. After extraction, the extracts were precipitated with three volumes of 95% (*v*/*v*) ethanol overnight at 4 °C. Subsequently, the precipitations were redissolved, dialyzed for 3 days (Dialysis membrane, molecular weight cut off: 3.5 kDa, Solarbio, Beijing, China), freeze-dried, and stored at −20 °C.

### 3.3. Isolation and Purification of OPPs

OPPs were purified by using a DEAE Cellulose-52 column (5 × 50 cm). Briefly, OPPs were dissolved in 40.0 mL of deionized water at the concentration of 25.0 mg/mL and filtered through a 0.45 µm syringe filter (JinTeng Company, Tianjin, China). The solution was then loaded onto a DEAE Cellulose-52 column and successively eluted by the deionized water and the 0.1 mol/L of NaCl solution at a flow rate of 1.0 mL/min. Each tube (5 mL/tube) was checked at 490 nm by the phenol-sulfuric acid method. Finally, one purified polysaccharide fraction named OPP-D was obtained (about 0.62 g), and the purity of OPP-D was checked by high-performance size-exclusion chromatography.

### 3.4. Structural Characterization of OPP-D

#### 3.4.1. Chemical Composition Analysis

The contents of total polysaccharides, total proteins, and total uronic acids of OPP-D were evaluated by the phenol-sulfuric acid assay, Bradford’s method, and the *m*-hydroxydiphenyl assay, respectively [11].

#### 3.4.2. Determination of Constituent Monosaccharides

Constituent monosaccharides of OPP-D were investigated by HPLC analysis (U3000, Thermo Fisher Scientific, Waltham, MA, USA) based on the previous method [11]. Ara (0.25 M), Gal (0.25 M), GalA (0.5 M), Glc (0.5 M), GlcA (0.5 M), Man (0.5 M), Rha (0.5 M), and Xyl (0.25 M) were mixed and used as a mixture standard solution.

#### 3.4.3. Fourier Transform Infrared Spectroscopy Analysis

The Fourier transform infrared (FT–IR) spectroscopy analysis of OPP-D was also investigated by the previous method [11]. The Nicolet iS 10 FT–IR (Thermo Fisher Scientific, Waltham, MA, USA) was used for the determination of the IR spectra of OPP-D in the frequency range of 4000–400 cm^−1^. The DE of OPP-D was also evaluated from FT–IR spectra on the basis of the previous methods [11,19].

#### 3.4.4. NMR Analysis

The OPP-D sample (20 mg) was dissolved in 0.5 mL of D_2_O overnight for NMR analysis. 1D NMR spectra, including ^1^H and ^13^C analyses, were recorded on a Bruker Ascend 600 MHz spectrometer (Bruker, Rheinstetten, Germany) with a z-gradient probe with proton and carbon frequencies of 600.13 and 150.90 MHz, respectively.

#### 3.4.5. Determination of Molecular Weight and Particle Size

The purity, absolute molecular weight (*M_w_*), polydispersity (*M_w_*/*M_n_*), and radius of gyration (*R_g_*) of OPP-D were determined by high-performance size-exclusion chromatography coupled with multi-angle laser light scattering and refractive index detector (HPSEC-MALLS-RID, Wyatt Technology Co., Santa Barbara, CA, USA) on the basis of the previous method [9].

The hydrodynamic radius (*R_h_*) of OPP-D was measured by using a dynamic light scattering (DLS, Zetasizer Nano, ZEN3600, Malvern Instruments, UK) according to a previous study with some modifications [25]. The sample was dissolved in 0.9% of NaCl aqueous solution. The determination was carried out at a constant temperature of 25 °C and at a scattering angle of 90°. The wavelength of the laser beam was set as 633 nm and the signal acquisition time was 5 s for 10 times.

#### 3.4.6. Determination of Apparent Viscosity

The apparent viscosity of OPP-D was measured by the previous method [9]. In brief, two concentrations (4.0% and 6.0%, *w*/*v*) of OPP-D were selected and dissolved in distilled water, respectively. The apparent viscosity was determined by a Discovery Hybrid Rheometer-1 (DHR-1, TA instruments, New Castle DE, USA) equipped with a 40 mm diameter parallel steel plate with a 1.0 mm gap. Flow curves of OPP-D were determined at 25 °C with the shear rate range from 0.01 to 100 s^−1^.

### 3.5. Evaluation of Bioactivities of OPP-D

#### 3.5.1. Determination of In Vitro Antioxidant Activities

Both ABTS and nitric oxide (NO) radical scavenging activities of OPP-D were evaluated by the formerly reported methods [11]. In brief, vitamin C was used as positive control. The ABTS and NO radical scavenging activities of OPP-D were evaluated at five different concentrations, respectively, and on the basis of a logarithmic regression curve, the IC_50_ values (mg/mL) of OPP-D could be determined.

#### 3.5.2. Determination of In Vitro α-amylase and α-glucosidase Inhibitory Activities

The inhibitory activities of OPP-D against α-amylase and α-glucosidase were evaluated by the previously reported methods [11]. The acarbose and distilled water were used as positive and blank controls for both α-amylase and α-glucosidase inhibitory activities. The α-amylase and α-glucosidase inhibitory activities of OPP-D were evaluated at five different concentrations, respectively, and on the basis of a logarithmic regression curve, the IC_50_ values (mg/mL) of OPP-D could be calculated.

#### 3.5.3. Determination of In Vitro Binding Properties

The in vitro binding properties of OPP-D, including fat, cholesterol, and bile acid-binding capacities, were evaluated by the previously reported methods [9,11]. In brief, for the investigation of fat and cholesterol-binding capacities of OPP-D, deionized water, and carboxymethyl cellulose were used as negative and positive controls, respectively. The fat binding capacity of OPP-D was expressed as milligram of binding fat per milligram of OPP-D (mg/mg). The cholesterol-binding capacity of OPP-D was expressed as milligram of binding cholesterol per gram of OPP-D (mg/g). Moreover, the cholestyramine was used as a positive control for the investigation of bile acid-binding capacity. The bile acid-binding capacity of OPP-D was expressed as a percentage of blank control (%).

#### 3.5.4. Determination of In Vitro Prebiotic Activity

The in vitro prebiotic activity of OPP-D was evaluated by a previous study with minor modifications [37]. Carbohydrate-free MRS broth was used as the basal medium to evaluate the in vitro prebiotic activity. Three Lactobacillus strains, including *L. acidophilus* CICC 6089, *L. rhamnosus* CICC 6133, and *L. rhamnosus* CICC 6151, were selected to determine whether OPP-D was the potential substrate for promotion of the growth of Lactobacillus strains. Inulin and the basal MRS were used as the positive and blank controls, respectively. The samples were prepared as concentrations of 1.0, 2.0, and 3.0% (*w*/*v*), filter-sterilized, and then added into the MRS broth. Each Lactobacillus strain was transferred into MRS broth medium at a concentration of 1 × 10^7^ CFU/mL. Afterwards, *L. acidophilus* CICC 6089 and *L. rhamnosus* CICC 6151 were incubated at 37 °C for 24 h, and *L. rhamnosus* CICC 6133 was incubated at 30 °C for 24 h, respectively. Finally, the optical density values of samples were measured at 600 nm.

SCFAs were determined by gas chromatography according to a previous study with minor modifications [38]. In brief, the fermented broths were centrifuged at 6000× *g* for 10 min. Then, 0.4 mL of supernatants were mixed with 0.4 mL of internal standard (0.05 M of 2-ethylbutyric acid), and filter through a 0.22 μm membrane filter. The mixed solution was analyzed by Agilent 7890 series GC system (Agilent Technologies, Palo Alto, CA, USA) with an HP-INNOWAX column (30 m × 0.25 cm × 0.25 μm, Agilent, USA).

### 3.6. Statistical Analysis

All experiments were conducted in triplicate, and data were expressed in means ± standard deviations. Statistical significances were carried out by one-way analysis of variance (ANOVA), taking a level of *p* < 0.05 as significant to Duncan’s multiple range test.

## 4. Conclusions

In this study, the chemical structure, chain conformation, and biological activities of a purified polysaccharide (OPP-D) were investigated. Results showed that OPP-D was a purified pectic-polysaccharide mainly composed of Rha, GalA, and Gal, and the proposed structure of OPP-D was Rha and GalA units as the backbone with galactan side chains. The chain conformation of OPP-D in aqueous solution was determined as an air-core sphere with branching chains for the first time. In addition, OPP-D exhibited remarkable in vitro antioxidant activities, anti-diabetic activity, and binding capacities, as well as prebiotic effect, which might be associated with its *M_w_*, DE, content of uronic acids, and highly branched globular conformation. Results suggested that OPP-D had great potential applications to be further explored in the food and medical industries. Based on the favorable effects of OPP-D on prebiotic activities, further studies about the effects of OPP-D on intestinal microorganisms through the in vitro fermentation model and in vivo mice model could be considered.

## Figures and Tables

**Table 1:** Effect of OPP-D on the growth and production of short-chain fatty acids of three probiotics after fermentation for 24 h.

Bacteria	Carbon Source	OD_600_	Total SCFAs (mM)
***L. acidophilus* CICC 6089**	Control	0.23 ± 0.01 ^d^	8.47 ± 0.98 ^d^
	1% OPP-D	0.28 ± 0.02 ^cd^	16.17 ± 1.53 ^d^
	2% OPP-D	0.31 ± 0.01 ^cd^	28.46 ± 2.27 ^c^
	3% OPP-D	0.35 ± 0.01 ^c^	31.32 ± 2.78 ^c^
	1% Inulin	0.47 ± 0.03 ^b^	30.34 ± 1.92 ^c^
	2% Inulin	0.63 ± 0.01 ^a^	41.99 ± 3.25 ^b^
	3% Inulin	0.65 ± 0.06 ^a^	51.56 ± 3.76 ^a^
***L. rhamnosus* CICC 6133**	Control	0.29 ± 0.01 ^c^	3.51 ± 0.45 ^e^
	1% OPP-D	0.33 ± 0.02 ^b^	10.28 ± 0.87 ^de^
	2% OPP-D	0.36 ± 0.01 ^b^	13.24 ± 2.16 ^cde^
	3% OPP-D	0.40 ± 0.01 ^a^	20.81 ± 1.85 ^bc^
	1% Inulin	0.35 ± 0.01 ^b^	16.94 ± 2.65 ^cd^
	2% Inulin	0.40 ± 0.00 ^a^	28.40 ± 2.79 ^b^
	3% Inulin	0.43 ± 0.00 ^a^	67.04 ± 6.04 ^a^
***L. rhamnosus* CICC 6151**	Control	0.24 ± 0.02 ^e^	6.36 ± 1.17 ^c^
	1% OPP-D	0.32 ± 0.01 ^d^	54.38 ± 4.38 ^b^
	2% OPP-D	0.33 ± 0.01 ^cd^	77.64 ± 6.13 ^a^
	3% OPP-D	0.40 ± 0.02 ^bc^	90.98 ± 10.52 ^a^
	1% Inulin	0.47 ± 0.01 ^b^	54.14 ± 3.56 ^b^
	2% Inulin	0.55 ± 0.01 ^a^	89.67 ± 6.76 ^a^
	3% Inulin	0.62 ± 0.05 ^a^	100.89 ± 8.57 ^a^

OPP-D, polysaccharide extracted by UAE and purified; SCFAs, short-chain fatty acids; Inulin was used as a positive control in prebiotic activity. Values represent mean ± standard deviation, and superscripts a–e differ significantly (*p* < 0.05) among OPPs. Statistical significances were carried out by ANOVA and Duncan’s test.
